# Will vaccination refusal prolong the war on SARS-CoV-2?

**DOI:** 10.1136/postgradmedj-2020-138903

**Published:** 2020-10-28

**Authors:** Robert Marcec, Matea Majta, Robert Likic

**Affiliations:** University of Zagreb School of Medicine, Zagreb, Croatia; University of Zagreb School of Medicine, Zagreb, Croatia; Department of Internal Medicine, University of Zagreb School of Medicine and University Hospital Centre Zagreb, Zagreb, Croatia

**Keywords:** Infectious diseases, Infection control, Public health, Public health, Clinical pharmacology

## Abstract

**Introduction:**

Severe acute respiratory syndrome coronavirus 2 (SARS-CoV-2) is a novel coronavirus that appeared in Wuhan, China in January 2020 and caused a global pandemic drastically changing everyday life. Currently, there are vaccine candidates in clinical trials and development, so it is only a matter of time before one is authorised for human use.

**Materials and methods:**

We collected public opinion survey results about attitudes towards SARS-CoV-2 vaccination conducted in 2020 in 26 European countries.

**Results:**

The pooled surveys were conducted on a total of 24 970 participants; on average only 58% (n=14 365/24 970) of responders across Europe were willing to get a SARS-CoV-2 vaccine once it becomes available, 16% (n=3998/24 970) were neutral, and 26% (n=6607/24 970) were not planning to vaccinate against SARS-CoV-2. Such a low vaccination response could make it exceedingly difficult to reach the herd immunity threshold for SARS-CoV-2 through vaccination.

**Conclusion:**

It is very important to start conducting educational public health activities on the topic of vaccination as soon as possible, before a vaccine becomes available, in order to improve attitudes towards SARS-CoV-2 vaccination. Only by educating the general public about the benefits, safety and efficacy of vaccines can we hope to avoid the unnecessary prolongation of the COVID-19 pandemic.

## INTRODUCTION

Severe acute respiratory syndrome coronavirus 2 (SARS-CoV-2) is a novel coronavirus that has emerged in Wuhan, China in January 2020 and has since spread worldwide and caused a global pandemic with 18 614 177 confirmed cases and 702 642 deaths accordion to the WHO situation report 199 from August 6, 2020.^1^ Our everyday lives have been changed drastically because of SARS-CoV-2 and the whole world is hoping for the development of a safe and effective vaccine. According to the WHO, 26 vaccine candidates are in the process of clinical trials, out of which 6 are already in phase III and 139 are in the process of preclinical evaluation.^2^ With such a large number of candidates, it is only a matter of time before a safe and effective SARS-CoV-2 vaccine is developed and authorised for worldwide human use. However, after vaccine development, we will be faced with an even more difficult challenge—convincing enough people to actually get vaccinated!

## MATERIALS AND METHODS

In August 2020, we conducted a search of the PubMed and Statista databases as well as the internet for survey results on the attitudes of the general population towards SARS-CoV-2 vaccination that were conducted in European countries on a nationally representative sample in 2020.

## RESULTS

**Figure 1:**
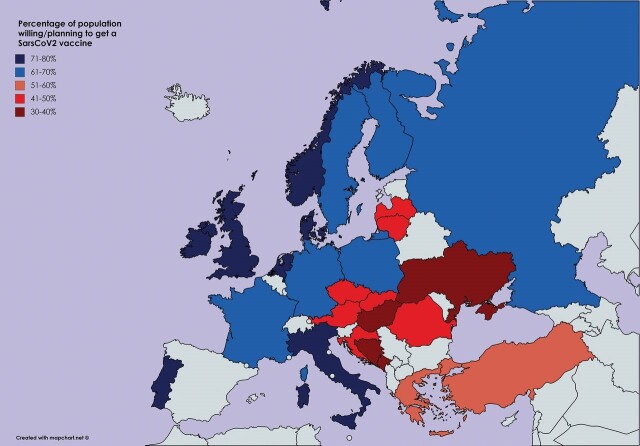
Map of Europe showing the percentage of participants per country who are willing/planning to take a SARS-CoV-2 vaccine according to collected public opinion surveys.^3–22^

Public opinion surveys on SARS-CoV-2 vaccination are a valuable tool for approximating the response rate to voluntary vaccination once a vaccine becomes available. We pooled available survey results regarding the public opinion on SARS-CoV-2 vaccination in 26 European countries conducted on a total of 24 970 participants, table 1.^3–22^ On average, only 58% (n=14 365/24 970) of participants of the collected surveys across Europe were willing/planning to get a SARS-CoV-2 vaccine once it became available, 16% (n=3998/24 970) were neutral and 26% (n=6607/24 970) were not willing/planning to vaccinate against SARS-CoV-2. Figure 1 shows the percentage of participants per country who were willing and/or planning to take a SARS-CoV-2 vaccine. The countries with the highest percentage of positive responses were: Denmark (80%; n=800/1000), the UK (79%; n=790/1000), Portugal (75%; n=750/1000), Italy (except Lombardy; 74%; n=740/1000), Ireland (73%; n=715/979), the Netherlands (73%; n=730/1000) and Norway (72%; n=844/1172). In contrast, the countries with the lowest percentage of positive responses included: Hungary (30%; n=300/1000), Bosnia and Herzegovina (32%; n=162/505), Montenegro (34%; n=278/819), Ukraine (37%; n=328/888), Slovakia (40,9%; n=409/1000), Romania (44%; n=452/1027) and Latvia (46%; n=368/800).

**Figure 2:**
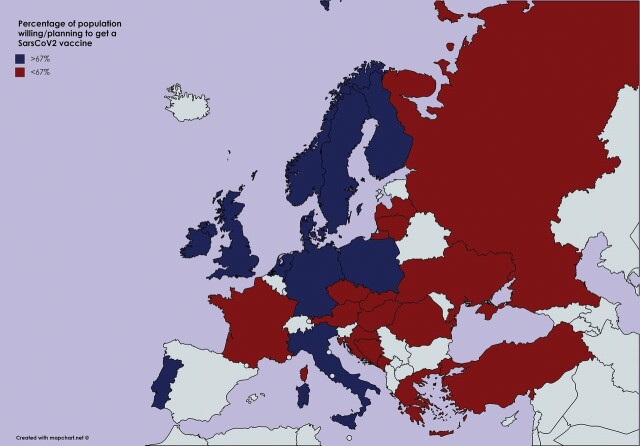
Map of Europe showing the counties which would/would not reach the herd immunity threshold (67%) through vaccination according to the collected public opinion survey results.^3–22^

As can be seen on figure 2, a significant number of European countries would not be able to reach the estimated herd immunity threshold (67%) even with a theoretically 100% effective SARS-CoV-2 vaccine utilisation, if the present trends in public opinion do not change by the time the vaccine becomes available.

## DISCUSSION

Due to the sheer number of potential SARS-CoV-2 vaccines in development, it is safe to presume that not one but many vaccines will be made available and approved in the near future, but even after safe and effective vaccines are developed, the war on SARS-CoV-2 will still be far from over. A plethora of practical and political problems like vaccine distribution and price formation will still remain to be resolved; however, it will all be futile if the general population refuses to participate in the widespread vaccination programmes. The goal of all vaccination programmes is to reach a certain proportion of sufficiently immune population members, without suffering the consequences of natural infection, where the whole population eventually ends up indirectly protected through herd immunity. When a sufficient percentage of population members is immune to the pathogen, the probability that still susceptible individuals encounter an infectious carrier is significantly decreased. The herd immunity threshold for SARS-CoV-2 is approximated to be 67% (R_0_ estimated at 3) and could potentially be reached through the natural course of the disease; however, the expected number of deaths to reach it would exceed 30 million people globally (IFR (infection fatality rate) of 0.6%).^23^ Serological studies in Spain demonstrated that the seropositivity in the general population was approximately 5%,^24^ implicating that Spain, a country which was a SARS-CoV-2 hotspot, is still far from reaching the satisfactory herd immunity levels through natural course of the disease and highlighting the importance of urgent vaccine development. The minimum percentage of the population which would have to be vaccinated in order to reach the herd immunity threshold depends mostly on the efficacy of the developed vaccine and will certainly have to be higher than the estimated 67% simply because no vaccine is 100% efficient (most childhood vaccines are effective in 85–95% of recipients.^25^)

The collected survey data presented in table 1 highlight a somewhat overlooked global problem of having a significant proportion of the population distrusting science and/or vaccines. If we hope to put an end to the SARS-CoV-2 pandemic through a widespread vaccination programme, it is important that we start conducting educational public health activities on the values and benefits of vaccination as soon as possible, before we even have a vaccine, in order to improve the attitudes towards upcoming widespread SARS-CoV-2 vaccination as much as possible. The impact of such educational interventions should not be limited only to the SARS-CoV-2 vaccination but should also entail highlighting the benefits of increased response rates to other vaccination programmes as well. In recent years, childhood vaccination rates have been falling,^26^ leading to a rise in preventable infectious disease outbreaks such as measles.^27^

**Table 1:** Results of public opinion surveys on the topic of SARS-CoV-2 vaccination willingness for 26 European countries

	Country	Negativeresponse %(n)	Neutral orno response %(n)	Positiveresponse %(n)	Number ofparticipants	Survey (*result publication) date	Source
1	Austria	34.3 (514)	18.2 (274)	47.5 (714)	1502	May 23–27, 2020	Eberl JM, Paul KT, Partheymüller J. Corona Blog [Internet]. Vienna: corona vaccination: reluctance among the population—but it should be free. [Accessed 6 August 2020]. Available at: https://viecer.univie.ac.at/corona-blog/corona-blog-beitraege/blog50/
2	Bosnia and Herzegovina	39 (197)	29 (146)	32 (162)	505	July 23–27, 2020	Klix. As many as 39% of B&H citizens do not plan to be vaccinated against COVID-19 [Internet]. [Accessed 6 August 2020]. Available at: https://www.klix.ba/vijesti/bih/cak-39-posto-gradjana-bih-ne-planira-se-vakcinisati-protiv-covida-19/200806026
3	Croatia	41.2 (577)	11.5 (161)	47.3 (662)	1400	23 May 2020*	Croatian Radiotelevision. CRO rating about coronavirus and opening borders before the season [Internet]. 23 May 2020. [Accessed 6 August 2020]. Available at: https://vijesti.hrt.hr/617756/hrejting-o-koronavirusu-i-otvaranju-granica-uoci-sezone
4	Czech Republic	43 (433)	8 (81)	49 (493)	1007	May 20–21, 2020	Stefan V, Mašková M, Český Rozhlas LV. Median: if there was a vaccine against COVID-19 today, only 49% of Czechs would be vaccinated. On the contrary, 43% would not ‘go’ [Internet]. 25 May 2020. [Accessed 6 August 2020]. Available at: https://plus.rozhlas.cz/median-kdyby-tady-dnes-byla-vakcina-proti-covidu-19-nechalo-se-ockovat-jen-49-8210262
5	Denmark	8 (80)	12 (120)	80 (800)	≈1000	April 2–15, 2020	Neumann-Böhme S, Varghese N, Sabat I, Barros P, Brouwer W, & van Exel J *et al* (2020). Once we have it, will we use it? A European survey on willingness to be vaccinated against COVID-19. The European Journal of Health Economics. 26 June 2020 doi: 10.1007/s10198-020-01208-6
6	Finland	22 (220)	9 (90)	69 (690)	1000	May 2020	Helsinki News. USU-gallup: the majority of Finns are sure to take a coronavirus vaccine [internet]. 29.5.2020. [Accessed 6 August 2020]. Available at: https://www.helsinginuutiset.fi/paikalliset/1859467
7	France	10 (100)	28 (280)	62(620)	≈1000	April 2–15, 2020	Neumann-Böhme S, Varghese N, Sabat I, Barros P, Brouwer W, & van Exel J, *et al* (2020). Once we have it, will we use it? A European survey on willingness to be vaccinated against COVID-19. The European Journal of Health Economics. 26 June 2020 doi: 10.1007/s10198-020-01208-6
8	Germany	10 (100)	20 (200)	70 (700)	≈1000	April 2–15, 2020	Neumann-Böhme S, Varghese N, Sabat I, Barros P, Brouwer W, & van Exel J, *et al* (2020). Once we have it, will we use it? A European survey on willingness to be vaccinated against COVID-19. The European Journal of Health Economics. 26 June 2020 doi: 10.1007/s10198-020-01208-6
9	Greece	34.1 (294)	13.8 (119)	52.1 (450)	863	July 20–21, 2020	News24/7. How many Greeks would not take a coronavirus vaccine if it was available [Internet]. 23 July 2020. [Accessed 6 August 2020]. Available at: https://www.news247.gr/20-20/posoi-ellines-den-tha-ekanan-emvolio-koronoioy-an-itan-diathesimo.7684851.html
10	Hungary	55 (550)	15 (150)	30 (300)	1000	February 10–12, 2020	Index in Hungary. ‘If a vaccine against coronavirus (COVID-19) is developed, will you take it?’ Chart. [Internet]. 14 February 2020. Statista [Accessed 6 August 2020]. Available at: https://www.statista.com/statistics/1103050/hungary-attitude-towards-vaccine-against-the-coronavirus/
11	Ireland	17 (166)	10 (98)	73 (715)	979	June 15–30, 2020	The Irish Pharmaceutical Healthcare Association. COVID-19: patient attitudes assessed in Ireland [internet] 14 July 2020. [Accessed 6 August 2020]. Available at: https://mapbiopharma.com/home/2020/07/COVID-19-patient-attitudes-assessed-in-ireland/
12	Italy (except Lombardy)	7 (70)	19 (190)	74 (740)	≈1000	April 2–15, 2020	Neumann-Böhme S, Varghese N, Sabat I, Barros P, Brouwer W, & van Exel J, *et al* (2020). Once we have it, will we use it? A European survey on willingness to be vaccinated against COVID-19. The European Journal of Health Economics. 26 June 2020 doi: 10.1007/s10198-020-01208-6
13	Latvia	39 (312)	15 (120)	46 (368)	800	1 June 2020*	Skaties. Almost half of those surveyed would use the COVID-19 vaccine [internet]. 1 June 2020. [Accessed 6 August 2020]. Available at: https://skaties.lv/zinas/latvija/sabiedriba/gandriz-puse-aptaujato-izmantotu-vakcinu-pret-COVID-19/
14	Lithuania	38 (205)	15 (81)	47 (253)	539	June 5–8, 2020	Levickytė P. Lithuanian National Radio and Television. Poll: most residents support ending the quarantine but are in no hurry to attend the events [Internet]. 15 June 2020. [Accessed 6 August 2020]. Available at: https://www.lrt.lt/naujienos/lietuvoje/2/1188609/apklausa-dauguma-gyventoju-pritaria-karantino-nutraukimui-taciau-i-renginius-neskuba
15	Montenegro	55 (450)	11 (90)	34 (278)	819	May 26–28, 2020	Attitudes of montenegrin citizens about the coronavirus [Internet]. 28 May 2020. [Accessed 6 August 2020]. Available at: https://www.Unicef.org/montenegro/sites/Unicef.org.montenegro/files/2020-06/IPSOS%2028.%20maj%20zdravlje%20-%20za%20sajt.pdf
16	Netherlands	8(80)	19 (190)	73 (730)	≈1000	April 2–15, 2020	Neumann-Böhme S, Varghese N, Sabat I, Barros P, Brouwer W, & van Exel J, *et al* (2020). Once we have it, will we use it? A European survey on willingness to be vaccinated against COVID-19. The European Journal Of Health Economics. 26 June 2020 doi: 10.1007/s10198-020-01208-6
17	Norway	7 (82)	21 (246)	72 (844)	1172	4 June 2020*	Research Council of Norway. Population attitudes towards vaccines (PPT). [Internet]. 4 June 2020 [Accessed 13 August 2020]. Available at: https://www.forskningsradet.no/contentassets/96ad9ee96f7a460cada4501ad0b3502d/vaksinetall.pptx
18	Poland	11 (56)	20 (103)	69 (355)	514	March 20–23, 2020	IMAS International. ‘If there was a coronavirus (COVID-19) vaccine, would you use it?’ Chart. [Internet]. 4 January 2020. Statista. [Accessed 6 August 2020]. Available at: https://www.statista.com/statistics/1111564/poland-attitude-towards-vaccine-for-the-coronavirus-COVID-19/
19	Portugal	5 (50)	21 (210)	75 (750)	≈1000	April 2–15, 2020	Neumann-Böhme S, Varghese N, Sabat I, Barros P, Brouwer W, & van Exel J, *et al* (2020). Once we have it, will we use it? A European survey on willingness to be vaccinated against COVID-19. The European Journal of Health Economics. 26 June 2020 doi: 10.1007/s10198-020-01208-6
20	Romania	33 (339)	23 (236)	44 (452)	1027	May 13–14, 2020	Romanian Institute for Evaluation and Strategy. ‘Would you be willing to be vaccinated against COVID-19 as soon as an approved vaccine was available?*.’ Chart. [Internet]. 18 May 2020. Statista. [Accessed 6 August 2020]. Available at: https://www.statista.com/statistics/1118531/romania-willingness-to-be-vaccinated-against-COVID-19/
21	Russia	24 (240)	13 (130)	63 (630)	1000	24 April 2020	Platforma, und Online Market Intelligence, und Various sources (Google Drive). ‘Public opinion on vaccination against the coronavirus (COVID-19) in Russia in April 2020.’ Chart. [Internet]. 29 April 2020. Statista. [Accessed 6 August 2020]. Available at: https://www.statista.com/statistics/1114599/opinion-on-vaccination-against-COVID-19-in-russia/
22	Slovakia	27.9 (279)	31.2 (312)	40.9 (409)	1000	April 21–23, 2020	Horak O, Denník N. New survey: less than half of Slovaks could be vaccinated against coronavirus [Internet]. 7 May 2020. [Accessed 6 August 2020]. Available at: https://dennikn.sk/1884358/novy-prieskum-ockovat-na-koronavirus-by-sa-dala-menej-ako-polovica-slovakov/
23	Sweden	18 (74)	15 (61)	67 (274)	409	July 9–13, 2020	Lundqvist A, Sifo K, Orbe J. Kantar: report on trust, anxiety and behaviour during the corona crisis [Internet]. 13 July 2020. [Accessed 6 August 2020]. Available at: https://www.msb.se/contentassets/7097cd4415d04ecc919dc07d0fb157bb/pdf-msb-resultat-coronaundersokning-20200713.pdf
24	Turkey	44.2 (679)	–	55.8 (858)	1537	11 March 2020*	The Turkey Report. If a coronavirus vaccine is found, would you get it? [Internet]. 11 March 2020. [Accessed 6 August 2020]. Available at: https://www.turkiyeraporu.com/korona-virusu-asisi-bulunsa-yaptirir-misiniz
25	UK	6 (60)	15 (150)	79 (790)	≈1000	April 2–15, 2020	Neumann-Böhme S, Varghese N, Sabat I, Barros P, Brouwer W, & van Exel J, *et al* (2020). Once we have it, will we use it? A European survey on willingness to be vaccinated against COVID-19. The European Journal of Health Economics. 26 June 2020 doi: 10.1007/s10198-020-01208-6
26	Ukraine	45 (400)	18 (160)	37 (328)	888	July 2020	Assessment of the COVID-19 threat in Ukraine [Internet]. 24 July 2020 [Accessed 6 August 2020]. Available at: https://www.ipsos.com/en-ua/assessment-COVID-19-threat-ukraine
	Total	26 (6 607)	16 (3 998)	58 (14 365)	24 970		

It is worrisome that on average only 58% (n=14 365/24 970) of the surveyed Europeans were willing to get a SARS-CoV-2 vaccination once it becomes available and that 26% (n=6607/24 970) would refuse to take it altogether. Such public opinion will impede reaching herd immunity levels through vaccination, however, since approximately 16% (n=3998/24 970) of the surveyed remained undecided, a widespread public intervention aimed at increasing the awareness of importance, safety and efficacy of vaccines in control of this and many other infectious diseases could be worthwhile.

Although some may argue that Russia is not a European country, we have decided to include it in our survey pool on the basis that the European part of Russia occupies 39% of Europe’s total land area and although this portion comprises only 25% of Russia’s whole land territory, it houses 78% of the Russian population.

It is also important to understand the reasons why people do not want to get vaccinated. Among the participants of the survey that was conducted in Russia who stated that they would not get a vaccine, 19% stated that they feel no need for it, 18% were concerned about the effectiveness of such a vaccine, 11% were not clearly informed about the new vaccine, 9% regarded the process of vaccination as dangerous, 9% stated the vaccines’ impact on the immune system as a reason not to get vaccinated, 8% did not believe in or wanted to get a vaccine without additional information, 5% did not believe in the existence of SARS-CoV-2, 5% were afraid of vaccination consequences, 4% stated contraindications for vaccination, 4% did not believe that SARS-CoV-2 is dangerous to them and 3% believed they had a good immune system.^28^ In contrast, a recent United States study reported a 67% acceptance rate for a COVID-19 vaccine, however with noticeable demographic and geographical disparities in vaccine acceptance.^29^

All the stated reasons emphasise the lack of proper information and trust towards science and vaccination among the members of the general public. Thus, scientists, physicians, public health and government authorities should all strive to address the questions and doubts of the general public and begin providing answers and reassurance in a clear, repetitive and organised way.

Global politics play an important role in the development of a SARS-CoV-2 vaccine and one could say that it is the equivalent of the 20th-century space race with governments working as quick as possible to be the first to develop a vaccine, motivated only by the victory in the vaccination race, and not necessarily by the prospect of saving lives. In our view, the global scientific community must not be subjected to political influence and it should remain transparent, honest and selfless. In a race for a vaccine, it is not unimaginable that some may sidestep important safety trials thus potentially endangering their citizens. One potential example of such a rushed vaccination approval is the Russian Sputnik vaccine, which was approved in Russia without a proper phase III trial. Such hurried and politicised vaccines have the potential to further undermine the trust in science and vaccine development and eventually do more harm than good.

One of the main limitations of this study is the fact that the public opinion studies for each evaluated country were conducted at different time points in 2020. The extremely small positive response rate in Hungary could be significantly influenced by the fact that the data were collected in February 2020, before the first Hungarian COVID-19 case was confirmed on March 4. The collected survey results represent the public’s opinion only in a certain period of time in which they were performed and can have a limitation of selection bias. The global situation regarding SARS-CoV-2 is not static, thus, the ever-increasing numbers of confirmed cases and fatalities surely have an impact on the public opinion towards SARS-CoV-2 vaccination.

The exact plans of the suggested public health interventions will have to differ from country to country. The unfortunate global success of the anti-vaccination movement relies heavily on social media^30^, which provide excellent and cheap means for widespread misinformation distribution; thus, it seems reasonable to also rely on mass media and online social platforms to distribute the positive messages regarding the vaccination benefits. It would also be key to review why the already performed educational campaigns on this topic seem to have failed so far.

Like often in science, our study results raise more questions and potential problems than answers. As can be seen, the percentage of people willing to vaccinate against SARS-CoV-2 varies significantly from country to country, ranging from as little as 30% to 80%. The question that remains is whether neighbouring countries with different percentages of vaccinated people will still insist on a negative PCR test or proof of vaccination status for free travel in the future? Will there be significant differences among the developed vaccines (like their efficacy, multiple-dose requirements, length of protection and potential side effects) which could influence the general response to vaccination? If the vaccination response remains too low, will we have to quickly change plans and start investing financial and scientific resources more into research of the treatments for COVID-19 or should we have done so from the start in parallel to vaccine development? The list goes on and on, but the only thing that is certain for now is that reaching adequate SARS-CoV-2 vaccination levels globally will be one of the most difficult challenges the health community has faced so far and that the appropriate time to act is now. Only by educating the public about the benefits, safety and efficacy of vaccines can we hope to avoid the prolongation of the COVID-19 pandemic even after vaccines become widely available.

**Main messages**

- Our pooled survey results reveal that on average only 58% of responders across Europe were willing to get a SARS-CoV-2 vaccine once it becomes available.
- Low vaccination response could make reaching herd immunity to SARS-CoV-2 difficult and unnecessarily prolong the pandemic.
- We propose the start of educational public health activities on the topic of vaccination as soon as possible, before the vaccine becomes available, in order to improve attitudes towards SARS-CoV-2 vaccination.

**Current research questions**

- Why is vaccination hesitancy to SARS-CoV-2 so high in Europe?
- What public health actions would be most effective in influencing public opinion regarding vaccination?
- Has the general public’s attitude towards SARS-CoV-2 vaccination changed since this study was conducted?

**What is already known on the subject**

- In recent years, vaccine hesitancy has been on the rise, with childhood vaccination rates declining and preventable diseases outbreaks occurring more frequently.
- Once one or more SARS-CoV-2 vaccines become available, reaching adequately high vaccination levels in the population will be critical for achieving herd immunity.
- Low vaccination rates against SARS-CoV-2 could have serious medical, social and economic consequences by extending the duration of the pandemic.
